# Generating Contextual Variables From Web-Based Data for Health Research: Tutorial on Web Scraping, Text Mining, and Spatial Overlay Analysis

**DOI:** 10.2196/50379

**Published:** 2024-01-08

**Authors:** Pablo Galvez-Hernandez, Angelina Gonzalez-Viana, Luis Gonzalez-de Paz, Ketan Shankardass, Carles Muntaner

**Affiliations:** 1 Lawrence S Bloomberg Faculty of Nursing University of Toronto Toronto, ON Canada; 2 Institute of Health Policy, Management and Evaluation Dalla Lana School of Public Health University of Toronto Toronto, ON Canada; 3 Public Health Agency of Catalonia Health Department Barcelona Spain; 4 Primary Healthcare Transversal Research Group Institut d’Investigacions Biomèdiques August Pi i Sunyer Barcelona Spain; 5 Consorci d'Atenció Primària de Salut Barcelona Esquerra Barcelona Spain; 6 Department of Heath Sciences Wilfrid Laurier University Waterloo, ON Canada; 7 MAP Centre for Urban Health Solutions Li Ka Shing Knowledge Institute St Michael’s Hospital Toronto, ON Canada; 8 Dalla Lana School of Public Health University of Toronto Toronto, ON Canada

**Keywords:** web scraping, text mining, spatial overlay analysis, program evaluation, social environment, contextual variables, health assets, social connection, multilevel analysis, health services research

## Abstract

**Background:**

Contextual variables that capture the characteristics of delimited geographic or jurisdictional areas are vital for health and social research. However, obtaining data sets with contextual-level data can be challenging in the absence of monitoring systems or public census data.

**Objective:**

We describe and implement an 8-step method that combines web scraping, text mining, and spatial overlay analysis (WeTMS) to transform extensive text data from government websites into analyzable data sets containing contextual data for jurisdictional areas.

**Methods:**

This tutorial describes the method and provides resources for its application by health and social researchers. We used this method to create data sets of health assets aimed at enhancing older adults’ social connections (eg, activities and resources such as walking groups and senior clubs) across the 374 health jurisdictions in Catalonia from 2015 to 2022. These assets are registered on a web-based government platform by local stakeholders from various health and nonhealth organizations as part of a national public health program. Steps 1 to 3 involved defining the variables of interest, identifying data sources, and using Python to extract information from 50,000 websites linked to the platform. Steps 4 to 6 comprised preprocessing the scraped text, defining new variables to classify health assets based on social connection constructs, analyzing word frequencies in titles and descriptions of the assets, creating topic-specific dictionaries, implementing a rule-based classifier in R, and verifying the results. Steps 7 and 8 integrate the spatial overlay analysis to determine the geographic location of each asset. We conducted a descriptive analysis of the data sets to report the characteristics of the assets identified and the patterns of asset registrations across areas.

**Results:**

We identified and extracted data from 17,305 websites describing health assets. The titles and descriptions of the activities and resources contained 12,560 and 7301 unique words, respectively. After applying our classifier and spatial analysis algorithm, we generated 2 data sets containing 9546 health assets (5022 activities and 4524 resources) with the potential to enhance social connections among older adults. Stakeholders from 318 health jurisdictions registered identified assets on the platform between July 2015 and December 2022. The agreement rate between the classification algorithm and verified data sets ranged from 62.02% to 99.47% across variables. Leisure and skill development activities were the most prevalent (1844/5022, 36.72%). Leisure and cultural associations, such as social clubs for older adults, were the most common resources (878/4524, 19.41%). Health asset registration varied across areas, ranging between 0 and 263 activities and 0 and 265 resources.

**Conclusions:**

The sequential use of WeTMS offers a robust method for generating data sets containing contextual-level variables from internet text data. This study can guide health and social researchers in efficiently generating ready-to-analyze data sets containing contextual variables.

## Introduction

### Background

Contextual variables refer to the social or physical attributes of geographic or jurisdictional areas (eg, country, city, neighborhood, and administrative health area) that are not derived from the characteristics of their members [1]. Common examples include social cohesion [2], social capital [3], and presence of green spaces [4]. Contextual variables have multiple applications in health and social research. As people living in the same community or context are likely to be exposed to a similar environment, contextual variables can be used in multilevel models to explain variability in health outcomes [5].

Although information on some contextual variables, such as census data, is widely available, accessing context-level data in emerging research fields can pose significant challenges. For example, monitoring systems may not exist yet to fully capture the social determinants of health (SDOH) across delimited areas. In addition, there may not be data available on the exposure and implementation of large-scale interventions targeting SDOH, making program and implementation evaluation studies challenging or impossible [6]. This could be the case for regional or state public policies and public health programs, such as provincial public health programs to promote local intersectoral collaborations to tackle SDOH [7] or national legislation to promote healthy nutrition to prevent obesity [8]. As these policies and programs can be implemented without an evaluation plan, and data might be complex or unavailable, they often remain unevaluated [9].

When structured databases or primary data gathering are not feasible, the internet can be a valuable resource for compiling information to define contextual variables. However, this presents several challenges: internet data are often cluttered, fragmented, and spread over multiple websites [10]. Moreover, the content of most websites is not designed for use by health researchers nor is it grouped by relevant contextual areas. To overcome these challenges, we developed a novel 8-step method, which we have termed web scraping, text mining, and spatial overlay analysis (WeTMS) to collect large amounts of internet data from websites, transforming it into meaningful data sets containing research-relevant variables, and classifying them based on delimited geographical or jurisdictional areas.

This method combines the techniques used in web scraping, text processing and mining, and spatial analysis. Web scraping, also known as web data mining, involves the creation of programs that can automatically download, parse, organize, and store information collected from the web in structured data sets [11]. This process is more efficient and less prone to errors compared with the traditional and laborious process of manually copying and pasting internet information into a spreadsheet [11]. Web scraping has been gaining traction in health research, fueling the rise of *infodemiology*, which analyzes the spread and impact of web-based information to inform public health and policy [12]. As of January 2023, a search of the keyword “web scraping” in Medline yielded 105 records, 95 of which were published starting from 2019. Articles using web scraping in health and social research mostly used information from social media [13,14] (eg, Twitter, Instagram, and TikTok), forums [15,16], business and review websites [17], and news web pages [18].

Similarly, text mining has been increasingly applied in health and social research [19]. Text mining is the process of extracting meaningful information from large volumes of unstructured text data using techniques such as text classification, sentiment analysis, and pattern recognition [19]. Examples include using sentiment analysis on social media posts to identify health and mental well-being issues [20] and characterizing mental health problems [21]. In addition, topic modeling has been used to understand public perceptions of the COVID-19 pandemic on Twitter [22] and to uncover health-related topics on social media [23].

Spatial overlay analysis is a group of methodologies used in geographic information systems to simultaneously display multiple layers of spatial information and assess the relationships between different geographic features and attributes [24]. Spatial overlay analysis can be used to examine the relationships between multiple layers of geospatial data to locate spatial points (eg, coordinates) in delimited geographic or jurisdictional areas. Geographic information system methods have been used in health geography and environmental epidemiology to study the geographic incidence or distribution of diseases [25].

When census data or data sets containing contextual variables are unavailable, researchers may have to engage in laborious manual extraction of web-based data, which can be time-consuming and susceptible to inaccuracies [11]. Currently, there is a gap in the literature regarding methods that enable researchers to automatically convert large volumes of internet text information into meaningful, ready-to-analyze data sets containing contextual data. We propose that by combining techniques used in WeTMS, researchers can efficiently extract, process, and geolocate vast amounts of internet text data to produce structured data sets that encompass variables reflecting the contextual characteristics of specific geographic or jurisdictional areas.

### Objectives

The aims of this study are 2-fold. First, we outline the implementation of the WeTMS method through a research case, creating data sets with contextual variables on health assets that could improve social connections for older adults across various health jurisdictions in Catalonia, Spain. Second, we analyze these data sets to describe the characteristics and registration trends of these health assets by local stakeholders.

In this tutorial, we first introduce the WeTMS method and describe its application to a research case for compiling data sets of health assets that could enhance social connections among older adults in the health jurisdictions of Catalonia. These assets include activities and resources in the community that can facilitate social interaction, such as social activities, walking groups for retirees, libraries, and senior community centers [26].

Next, we use these new data sets to extract assets with the potential to foster older adults’ social connections and conduct a descriptive analysis to explore their characteristics and asset registration trends across jurisdictions. This analysis demonstrates the potential application of this method in program evaluation. In addition, we discuss the challenges that health and social researchers may face during the WeTMS process and provide resources and programming codes to facilitate its application in other areas of research.

## Methods

### Context and Data Sources

In 2015, the government of Catalonia launched the Assets and Health platform as a component of 2 provincial public health programs that aimed to promote intersectoral collaborations among health and nonhealth organizations to tackle complex public health issues, including older adults’ lack of social connections [7]. The Assets and Health platform (created by the Asturias Health Observatory and shared through the Spanish Community Health Alliance) is a search engine and repository where stakeholders from multiple local organizations can register community health assets [27]. Health assets are activities and resources within the community that contribute to maintaining the health and well-being of individuals and groups [28].

Health assets were registered as “activities” (time-bound initiatives and structured interventions, like arts and crafts or supervised walking outings) and “resources” (permanent community fixtures such as associations, parks, and civic centers). Once registered, each health asset is stored on an individual website detailing characteristics, such as its title, description, location, and target population. These individual websites are linked to a search engine, enabling stakeholders to locate assets available in their basic health areas (BHAs), which can be used in collaborative interventions to address public health problems. Each BHA in Catalonia is a local health jurisdiction that functions as an administrative unit within the Catalan healthcare system [29]. In urban settings, BHAs typically cover specific neighborhoods or districts, whereas in rural areas, they may span one or more municipalities, as determined by demographic, epidemiological, and accessibility considerations.

### Overview of the WeTMS Method

**Figure 1:**
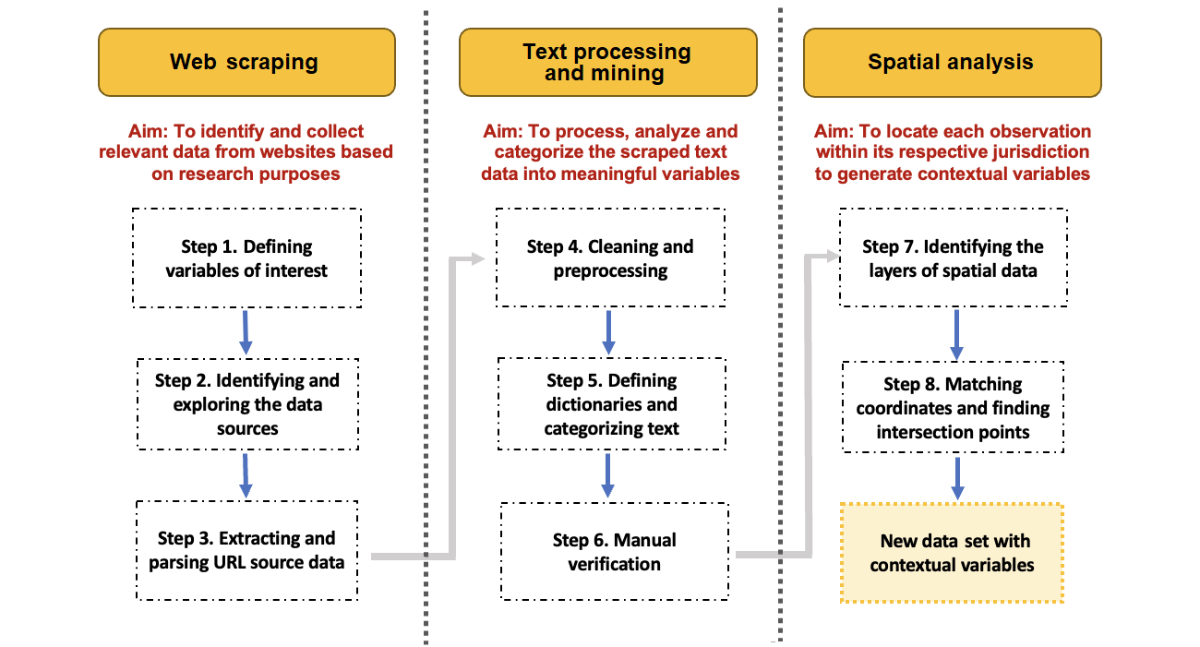
Overview of the web scraping, text mining, and spatial overlay (WeTMS) steps for generating contextual variables from unstructured web-based data.

The 8 steps of the proposed method are summarized in Figure 1. The first 3 steps involve identifying and extracting website data through web scraping, and then storing the information in structured data sets to facilitate their analysis. Steps 4-6 describe the application of text processing and mining techniques to analyze the scraped data, identify patterns in the text content, and classify the data into new variables and categories. Steps 7 and 8 elaborate on the use of spatial overlay analysis to locate data within delimited geographic or jurisdictional areas.

### Steps 1 to 3: Using Web Scraping for Data Extraction

#### Step 1: Defining Variables of Interest

The target information, including the type of data and the desired outputs for web scraping, was first outlined to avoid extracting irrelevant information. We aimed to generate context-level variables capturing the attributes and registration dates of community health assets to enhance social connections among older adults in Catalan BHAs. The data to be extracted from each website detailing a health asset included text elements such as title, description, target population, location, asset registration date, cost, duration, and activity topics. Other data types that could be targeted for extraction include images, links, and metadata, while outputs might be structured data sets such as CSV files, which contain prespecified variables.

#### Step 2: Identifying and Exploring the Data Sources

The second step involves identifying the URLs or web addresses containing the target information and understanding how their content is structured. Identifying URLs can present challenges such as information being dispersed across multiple websites or URLs being hidden or changing [30]. Consequently, sites may be missed because the web scraping “crawler” (the portion of code responsible for finding each URL) requires exact web addresses [30].

In our initial exploration of the Assets and Health search engine, only the last 100 registered health assets were displayed, and those URLs were hidden. A pattern in the URLs for websites describing each health asset, comprising a fixed segment and variable reference number, was identified using Chrome DevTools for network inspection.

The source code of the target websites, usually HTML, was examined to discern their organization and structure. The attributes of HTML elements containing target data (eg, asset titles and descriptions) were identified and used to program the web scraper. Additional information and resources required to implement step 2 are available in Multimedia Appendix 1 [11,31-36].

#### Step 3: Extracting and Parsing URL Source Data

After identifying the relevant URLs and HTML elements, a web scraper comprising a crawler, parser, and data handler was developed using Python 3.10 [37] on the PyCharm 2022.2.2 environment, and the libraries “requests” [38], “beautifulsoup4” [39], and “pandas” [40]. The web scraper also incorporated error-handling mechanisms to manage potential issues, such as connection failures when URLs were nonexistent. The code is explained in detail in Multimedia Appendix 2 and is publicly accessible on GitHub [41].

The web crawler requested 50,000 URLs from websites linked to the Assets and Health platform, comprising 25,000 activities and 25,000 resources, to capture reference numbers from the onset of the program, from July 2015 to December 23, 2022, which was the day when the data were scraped. The program “parser” then analyzed the HTML code of each existing URL and extracted the desired elements, stripping the text, which was automatically stored in 2 CSV data sets for activities and resources. The encoding of the data sets was revised to avoid mismatches between the character set used to represent the text data and that of the scraped text, as this can result in certain characters being displayed as symbols. An initial review of scrapped health assets was conducted to exclude irrelevant observations. We filtered out assets registered outside Catalonia or targeted solely at children and youth before proceeding with the text processing and mining steps.

### Steps 4 to 6: Text Processing and Mining to Generate Meaningful Contextual Variables

#### Step 4: Cleaning and Preprocessing

Cluttered and inconsistent text data obtained from web scraping were preprocessed for analysis [31]. We used RStudio (version 2022.12.0), with the “tm” [42] and “qdap” [43] libraries. The “tm” library provides functions for cleaning, preprocessing, and analyzing text data. The “qdap” library allows text categorization, word frequency calculation, tokenization, and clustering.

The first author manually examined a set of activities and resources from scraped text to assess the quality and structure of the text data. This step was crucial for identifying inconsistencies, such as assigning different age ranges (eg, 60 and 65 years) to older adults simultaneously.

To preprocess the text data, the columns containing free text, namely titles and descriptions of the health assets, were merged and converted to “corpus” objects—a data structure for text data in R. Columns with text derived from fixed responses were not preprocessed. Irrelevant stop words were then removed, text data were segmented into individual units that could be transformed into numerical variables (tokenization), and words were normalized to their root form (stemming) [44]. The 2 data sets, containing health assets registered as activities and resources, were processed independently. The code with explanations for this step can be accessed through GitHub [45].

#### Step 5: Defining Dictionaries and Categorizing Text

We used text mining techniques to develop a classification system to filter and categorize health assets for older adults to enhance social connections from all other registered activities and resources on the platform. Text classification is pivotal for the generation of new variables of interest from unstructured data, because it can categorize text into predefined classes or labels.

First, to classify health assets, we predefined new variables and categories created through a deductive approach, based on the literature on social connections [46,47]. We also used inductive processes to create new variables and categories based on patterns identified during the text analysis and discussion among the research team. Table 1 lists the new variables, categories, type of creation process, and literature sources. Detailed definitions of the new variables and categories are provided in Multimedia Appendix 3 [46,48-51].

Second, we created document-term matrixes from the corpus of preprocessed text data containing health asset titles and descriptions. A document-term matrix is a mathematical matrix that describes the frequency of terms in a collection of textual data [31]. The frequency of each word was calculated and sorted based on their frequency values, representing the number of times a term appeared in the title and description of health assets.

Third, over the course of 3 meetings, 2 researchers (PG-H and CM) identified and selected high-frequency words that were repeated 15 times or more in the scraped data, grouped them into topic-specific dictionaries, and refined the list. Eligibility criteria, informed by the definitions of each new variable category, were developed to determine which words to include in each dictionary.

Finally, a classification system was developed using a rule-based classifier. A rule-based classifier categorizes data into predefined classes by applying a set of human-defined rules and conditions based on the features and attributes of the data [52]. We opted for a rule-based system over more complex machine learning classifiers, as this approach is better suited for scenarios with a limited number of specific labels and smaller data sets and ensures efficiency and interpretability without the need for extensive training data [52,53]. Topic-specific dictionaries consist of lists of words related to the definitions of the predefined variable categories as conditions to classify the text data [54]. Finally, an R function was developed to automatically generate a new column for each new variable, search for dictionary words in the scraped data, and assign a new category value if a word was found. The classifier system, including topic-specific dictionaries, is accessible on GitHub [45].

**Table 1:** New variables and categories created for the classification system of health assets.

New variables		Categories within each variable	Source columns from scraped text data	Creation process and literature sources
**Activities**				
	Activity type	Leisure and skill development, physical activity, social facilitation, psychological therapies, awareness campaigns, health and social care, and befriending	Title and description	Deductive [46,48,49]
	Format	Group and individual	Title and description	Deductive [49]
	Focus	Direct and indirect	Title and description	Deductive [49]
	Age	Children, youth, adults, older adults, general population, minors unspecified, and adults unspecified	Description, target population, and topics	Deductive [50]
	Gender	Women, men, nonbinary, and any	Description, target population, and topics	Deductive [51]
	Vulnerable populations	Migrants, caregivers, substance use, physical diseases, risk social exclusion, mental diseases, and all^a^	Title, description, target population, and activity topics	Inductive
**Resources**				
	Resource type	Municipal natural and green space, health institution, social welfare institution, education institution, patient advocacy group, charitable and voluntary organization, faith-based organization, parent school associations, public library, civic center, sports institution, leisure and cultural association, neighborhood association, and cultural institution	Title and description	Inductive
	Focus	Direct and indirect	Title and description	Deductive [49]
	Age	Children, youth, adults, older adults, general population, minors unspecified, and adults unspecified	Title, description, and topics	Deductive [50]
	Gender	Women, men, nonbinary, and any	Description and topics	Deductive [51]
	Vulnerable populations	Migrants, caregivers, substance use, physical diseases, risk social exclusion, mental diseases, and all^a^	Title, description, and topics	Inductive

^a^“Substance use,” “physical diseases,” “risk social exclusion,” “mental diseases,” and “all” are simplified terms for target populations experiencing substance use, physical diseases, mental diseases, those at risk of social exclusion, and the general population.

#### Step 6: Manual Verification

The categories assigned to the new variables for each health asset were reviewed for inconsistencies by 2 researchers with expertise in the topic (PG-H and Angeli Chacaliaza). Manual verification refers to a one-by-one examination of the classified data by human reviewers to ensure the accuracy of the new variables created [55]. Verification involved an independent review of 200 health assets by 2 researchers to assess new variable categories based on eligibility criteria. Discrepancies were resolved through web meetings with manual reclassification if necessary. The data sets were then divided into groups of 500 health assets for independent review. Agreement rates between the verified and automatically generated variables were computed using the Excel software.

### Step 7 and 8: Spatial Overlay Analysis to Locate Observations

#### Step 7: Identifying the Layers of Spatial Data

We used a spatial overlay analysis to generate a new variable that identified the BHA in which each health asset was located. The analysis was conducted in RStudio (version 2022.12.0) because of its many packages specifically designed for spatial overlay analysis, such as “sp,” [56] “sf,” [57] “rgdal,” [58] “rgeos,” [59] and “ggplot2” [60].

In this step, 2 spatial layers were identified. The first layer consisted of polygonal data depicting 374 BHAs in Catalonia. The data were obtained from the open database of the General Directorate of Health Planning and Research in Catalonia. Polygonal data can represent geographic or jurisdictional regions by defining their boundaries [61]. The second layer comprises a vector of geographic point data for each health asset. Point data consisted of longitude and latitude coordinates obtained from the addresses scraped for each activity and resource using Excel add-on GeoCode, a map tool that uses Google services to automatically retrieve longitudes and latitudes from addresses.

#### Step 8: Matching Coordinate Reference System and Finding Intersection Points

Step 8 involves transforming the spatial data layers into a common coordinate reference system (CRS) and identifying the intersecting points. The CRS of a spatial object determines its location on the Earth’s surface. Thus, analyzing 2 or more spatial layers with different CRS can produce misleading outcomes [61]. To identify areas of overlap between health asset coordinates and BHAs, the following steps were taken: (1) coordinates were transformed to a simple feature object format, (2) simple feature objects were converted into single points using the “st_point” function, and (3) both spatial data layers were converted to a common CRS.

Finally, a spatial overlay analysis was performed using the “st_intersects” function to determine the BHA polygons with which each health asset point data intersected. The function was applied in a loop to each row of the activity and resource data sets. The resulting outputs are stored in new columns named “Code_BHA” and “Name_BHA.” The code, along with explanations for steps 7 and 8, is available in GitHub [45].

### Data Set Filtering and Descriptive Analysis

The new data sets were filtered using the new variables and categories to select health assets with the potential to foster social connections among older adults from all scrapped assets. Eligible health assets registered as activities and resources were included if (1) the target population included older adults, (2) the format was either group activities or individual activities fostering social connections (eg, befriending), and (3) they were located in Catalonia.

A descriptive analysis was conducted in RStudio (version 2022.12.0), to understand the characteristics and asset registration trends of stakeholders across BHAs. Frequencies and proportions were calculated for each category of the new variables (activity type, format, focus, age, sex, and vulnerable populations). Temporal registration trends of activities and resources were analyzed using time-series graphs with local polynomial regression fitting lines, a nonparametric method used to describe the deterministic variation in data [62]. We also computed the average weekly registration of activities and resources in each BHA, assuming a Poisson distribution, where λ represents the weekly health asset registrations per area. Finally, visualization techniques were used to analyze the temporal evolution of the registration of activities and resources on the Assets and Health websites across BHAs, as well as their geographic distribution.

### Ethical Considerations

The data collected in this study were publicly accessible and did not contain any personal or sensitive information. Thus, ethical approval and participant consent were not required for this study. In addition, before data collection, we verified that the websites of interest did not have any explicit prohibitions against automatic web scraping, such as a “robots.txt” file or similar declarations.

## Results

### Results From WeTMS

#### Web Scraping

Of the 50,000 URLs inspected, 17,305 contained websites describing health assets (9558 activities and 7747 resources) registered with local stakeholders from July 2015 to December 2022. The number of observations obtained through web scraping matched the total number of assets reported on the Assets and Health platform, thus demonstrating the efficacy of the web scraper. No missing values were detected for the main variables (eg, title, description, location, and date of asset registration). In the activity data set, 9.56% (480/5022) of observations did not disclose the *activity cost*, and 49.04% (2463/5022) did not report the *activity duration*. An example of an activity and resource, as they appear in the scraped data sets, is provided in Textbox 1.

**Example of a health asset registered as activity and resource extracted from the scraped text (English translation).**

**Activity row #860**

- Title: School for Adults
- Description: Reading and writing classes
- Population: Over 65 years old—anyone (district neighbors over 65 years old)
- Location: Campoamor Street 92, 08204, Civic Center Rogelio Soto, Sabadell, Barcelona, Catalonia, Spain
- Organizations: Civic Center Rogelio Soto, Campoamor Neighborhood Association
- Registration date: February 6, 2020
- Is free: Yes
- Categories: Women, older adults, people at risk of exclusion, school of health, mental health, or emotional well-being
- Time activity: From September 13, 2019, to June 30, 2020

**Resource row #1961**

- Title: Association of Retirees and Pensioners, La Pineda
- Description: Association that aims to promote cultural training and sports activities for older adults, as well as avoiding loneliness and social isolation, fostering relationships between them
- Registration date: May 15, 2017
- Location: Alfredo Kraus Street 20, 43481, La Pineda Vila-seca, Tarragona, Catalonia, Spain
- Categories: Older adults, mental health or emotional well-being, physical activity, community health

#### Text Mining

**Figure 2:**
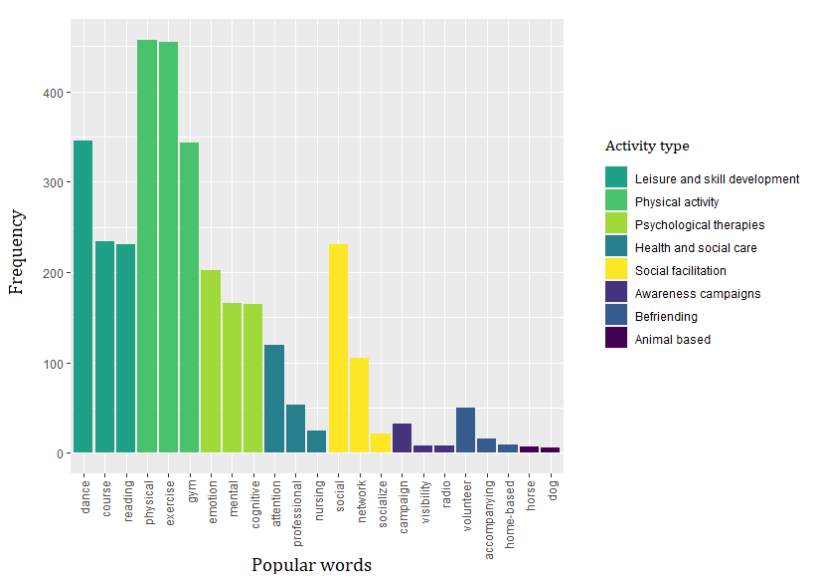
Categories within the “activity type” variable showcasing popular words derived from topic-specific dictionaries (English translation).

From the text processing of the corpus of titles and descriptions, a total of 12,560 tokens (or raw words) were identified for activities and 7301 for resources, of which 996 (7.9%) and 594 (8.1%) words had a frequency >15. Using words with a frequency of >15, we constructed 73 topic-specific dictionaries corresponding to each category of the new variables. For instance, for the *physical activity* category under the *activity type* variable, the topic-specific dictionary included words such as “physical,” “exercise,” “gym,” “yoga,” and “sport.” Figure 2 presents popular dictionary words for each category within the *activity type* variable.

After applying the rule-based classifier using topic-specific dictionaries, manual verification of the output yielded variable levels of agreement ranging from 62.02% (3417/5509) to 99.47% (4886/4912) across variables. For instance, variables with lower classification accuracy had a larger number of possible categories, a more evenly distributed number of observations across categories, or words repeated fewer than 15 times within the title and description corpus. The agreement rates between the verified and automatically generated databases are presented in Table 2.

**Table 2:** Agreement rate between manually verified and automatically classified data sets.

New variables generated			Correctly assigned categories, n (%)
**Activities data set (n=6260)**			
	Age	4855 (77.55)	
	Gender	6215 (99.28)	
	Vulnerable populations	5525 (88.26)	
	Activity type^a^	3417 (62.02)	
	Format^a^	5326 (96.67)	
	Focus^a^	5342 (96.97)	
**Resources data set (n=4912)**			
	Age	4029 (82.02)	
	Gender	4843 (98.59)	
	Vulnerable populations	3883 (79.05)	
	Resource type	3845 (78.28)	
	Focus	4886 (99.47)	

^a^Automatic classification of the categories for variables *activity type*, *format*, and *focus* was performed only for activities targeting older adults (n=5509).

#### Spatial Overlay Analysis

Coordinates for the locations of 0.36% (18/5055) of activities and 2.41% (109/4530) of resources were not identified. Manual searches on Google Maps using addresses allowed us to locate the coordinates for all but 7 activities and 2 resources. Through spatial overlay analysis, intersections between spatial points and BHAs were not identified for 26 activities or 4 resources. The newly generated columns for the variables *Code_BHA* and *Name_BHA* encompassed values for 318 distinct BHAs, representing 85% of the 374 BHAs.

### Results From Data Set Filtering and Descriptive Analysis

#### Filtering Health Assets With Potential to Enhance Older Adults’ Social Connections

**Figure 3:**
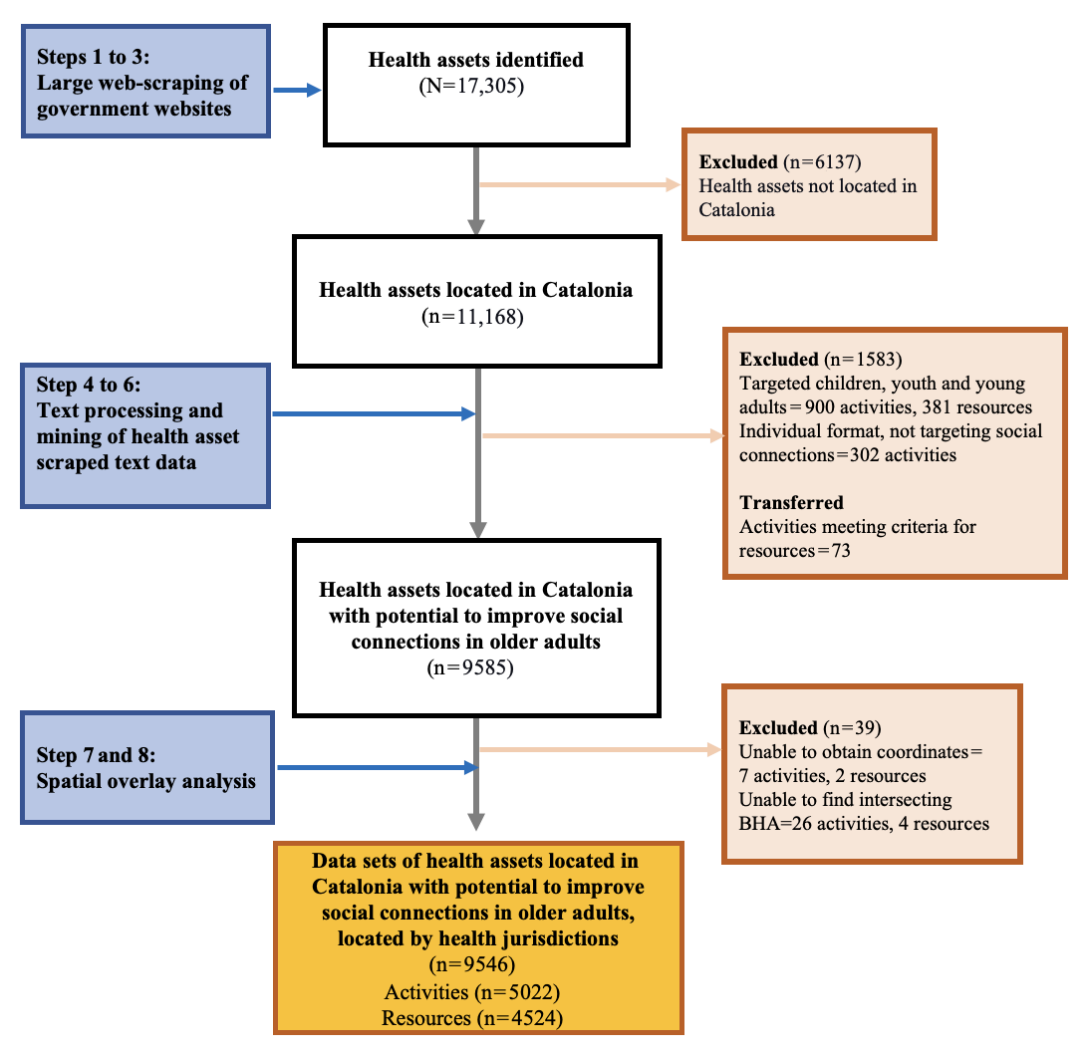
Flowchart for the filtering of health asset data sets by contextual variables related to social connection constructs generated using the web scraping, text mining, and spatial overlay (WeTMS) method. BHA: basic health area.

Using the newly generated contextual variables, we filtered the data sets of activities and resources to identify those with the potential to foster social connections among older adults. From the initial 17,305 health assets identified, we obtained 9546 eligible health assets, comprising 5022 activities and 4524 resources. The reasons for exclusion and the stages in which health assets were discarded are shown in Figure 3.

#### Characteristics of Eligible Health Assets

Of the health assets registered as activities, 24.59% (1235/5022) specifically targeted older adults, whereas 75.41% (3787/5022) targeted broader age ranges, including the older population. Most resources targeted the general population and included older adults, with only 4.12% (207/5022) being exclusively for older adults, such as civic centers for retirees. Only 2.49% (238/9546) of the health assets had a sex-specific target; these were predominantly women (n=212). Among all health assets, 13.5% (678/5022) of activities and 7.98% (361/4524) of resources were tailored for specific vulnerable groups, with physical or mental illness being the primary focus.

Group-oriented activities promoting social interactions accounted for 99.56% (5000/5022) of the eligible activities. However, only 4.36% (219/5022) explicitly used concepts related to social connections (eg, loneliness and social isolation) in titles and descriptions. Over 57% (2862/5022) of the activities were cost-free, and the most common activity duration was 1 to 3 months (975/5022, 19.41%). Data on format, duration, and cost are not available for resources.

**Figure 4:**
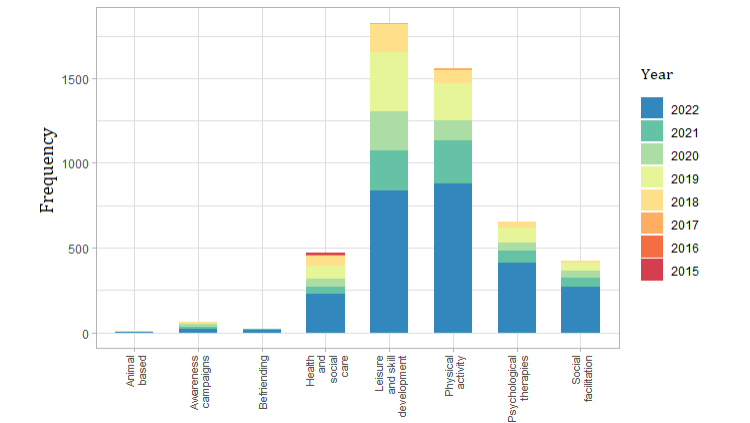
Number of activities with the potential to enhance older adults’ social connections by type and year of registration (2015-2022).

The analysis of the new variable *activity type* showed that leisure and skill development activities were most common (1844/5022, 36.72%). This included group handcrafts, dance, painting, theater, cooking, choir courses, and conversation groups that focused on shared-interest topics. Group exercise activities (eg, walking groups) accounted for 31.08% (1561/5022) of the activities. Over 22% (1103/5022) of the activities involved group activities with health and social professionals outside the health care center, including psychological therapies and health and social care. Finally, 8.46% (425/5022) were social facilitation activities such as group meetings to share common interests (eg, film forums). Overall, more than half of these activities were registered between 2021 and 2022 (Figure 4).

**Figure 5:**
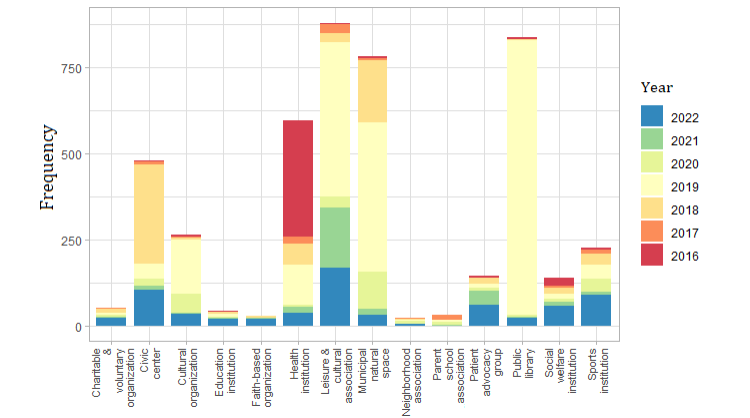
Number of resources with potential to enhance older adults’ social connections, by type and year of registration (2015-2022).

Almost 61.49% (2782/4524) of the registered resources facilitated exchange of knowledge and interests among older adults. These resources included leisure and cultural associations; public libraries; civic centers; and cultural, sports, and educational institutions. Municipal natural and green spaces where adults can gather accounted for 17.28% (782/4524) of the resources. A total of 595 (13.1%) health institutions and 140 (3.1%) social welfare institutions were found, including primary care centers, health and social foundations, and advocacy institutions promoting social inclusion. Other resources linked to health and social welfare include patient advocacy groups, faith-based organizations, and charitable and voluntary organizations. In contrast, most resources were registered before 2019 (Figure 5). A detailed descriptive analysis of the type, target population, focus, cost, format, and duration of health assets is included in Multimedia Appendix 4.

#### Overview of Registration Trends of Eligible Health Assets Across BHAs

**Figure 6:**
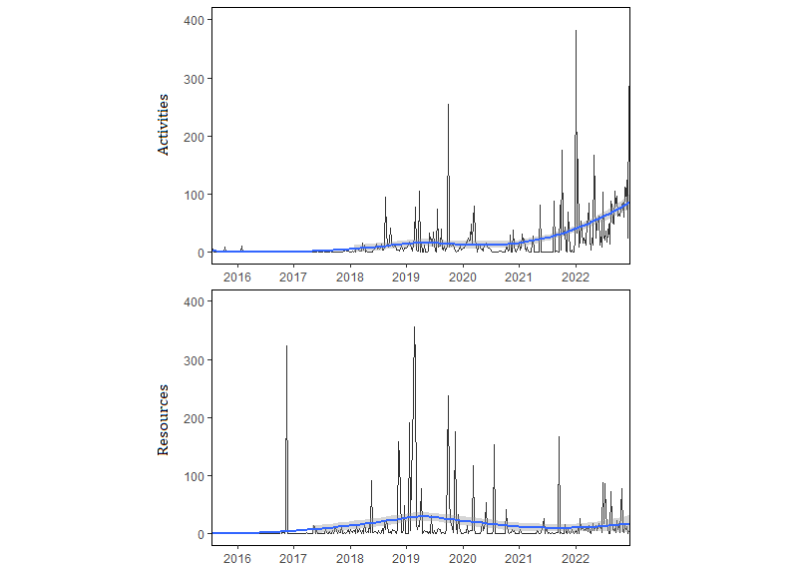
Weekly registration trends of health assets from July 2015 to December 2022. The y-axis represents the number of registered activities and resources per week. The x-axis represents time, labeled by years for clarity.

The first registry of a health asset on the Assets and Health websites occurred on July 23, 2015, and the last on December 23, 2022, the day when web scraping was conducted. Total registration of activities remained consistently low from the start of the program until early 2018, whereas for resources, a registration peak was observed in late 2016. Activity and resource registrations have increased from 2018 to mid-2020. A decline in registration was observed from early 2020 to mid-2021, coinciding with the outbreak of the COVID-19 pandemic. Local polynomial regression fitting lines showed a growing pattern in the registration of activities from 2021 onwards, whereas resource registration remained low (Figure 6).

On the basis of the observed trends, 4 implementation periods were defined to better understand registration trends: period 1, from July 2015 to January 2018; period 2, from February 2018 to February 2020; period 3, from March 2020 to May 2021; and period 4, from the end of June 2021 to December 2022. During the first two and a half years of the program, the average number of activities and resources registered per week across all BHAs was 0.37 and 3.47, respectively, increasing to 11.83 and 25.27 in period 2. During the COVID-19 pandemic in period 3, these figures decreased to an average of 3.19 activities and 20.64 resources per week. period 4 had the highest registration rate for activities (38.52/wk).

**Figure 7:**
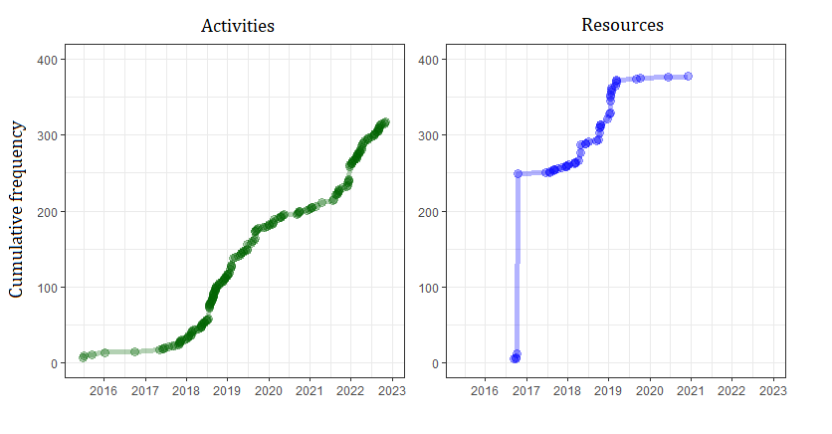
Cumulative frequency of basic health areas (BHAs) with registered health assets aimed at enhancing older adults’ social connections from July 2015 to December 2022. Each point represents a BHA at the time of its first asset registration on the Assets and Health platform, cumulative.

**Figure 8:**
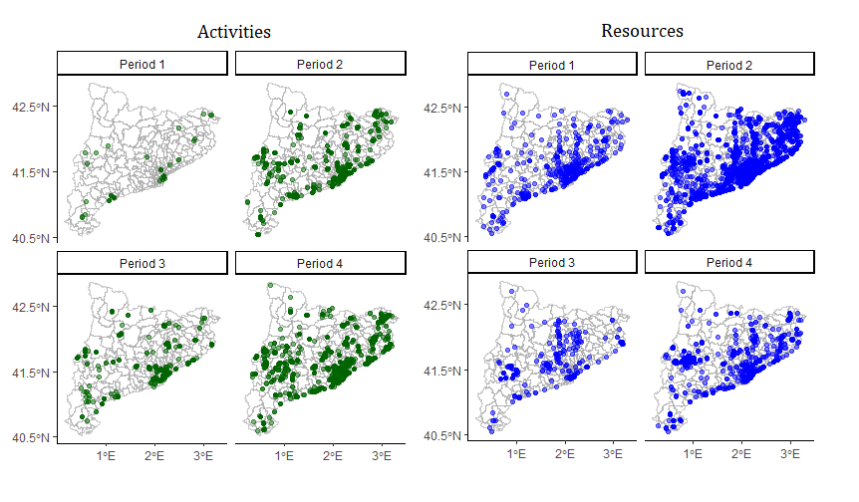
Geographic distribution of activities and resources with potential to enhance older adults’ social connections across basic health areas.

To calculate the registration trends in individual BHAs, we divided the number of BHAs with one or more activities registered by the total number of BHAs (n=374) for each period. We did not consider the resource data set because of the observed patterns suggesting centralized registration, rather than local registration. For instance, in late 2016, resources in 237 BHAs were registered in a single day. At the end of period 1, 8% (30/374) of the BHAs had one or more registered activities, which increased to 85% (318/374) by the end of period 4 (Figure 7; Table 3). The number of health assets registered per BHA varied significantly, ranging from 0 to 263 activities and 0 to 265 resources. The median number of activities registered per BHA from 2015 to 2022 was 5 (IQR 13.75) and 9 (IQR 10) for resources. Figure 8 illustrates the geographic distribution of the activities and resources registered in each period.

**Table 3:** Average number of health assets registered per week and proportion of basic health areas (BHAs) with one or more activities registered per period.

Time periods	Number of health assets		λ (95% CI)^a^			BHAs with registered activities	
	Activities	Resources	Activities	Resources	Values, n^b^		Cumulative proportion of BHA (%)^c^
Period 1: July 2015 to January 2018	50	462	0.37 (0.27-0.48)	3.47 (3.16-3.79)	30		8
Period 2: February 2018 to February 2020	1290	2755	11.83 (11.19-12.48)	25.27 (24.33-26.21)	154		49.2
Period 3: March 2020 to May 2021	562	301	8.64 (7.93-9.36)	4.63 (4.11-5.15)	28		56.6
Period 4: June 2021 to December 2022	3120	941	38.52 (37.16-39.87)	11.62 (10.87-12.36)	106		85

^a^λ denotes the average number of health assets registered per week in each period.

^b^Unique BHAs that registered activities targeting social connections in older adults for the first time in the specified period.

^c^BHAs that registered such activities up to and including each period with earlier registrations.

## Discussion

### Innovation: Generating Area-Specific Contextual Variables From Unstructured Web-Based Data

We introduce a novel approach for generating area-specific contextual variables from unstructured website data using WeTMS. By combining the methods commonly used in computer and data science, we were able to efficiently gather and transform large amounts of website data into comprehensive data sets of theoretically informed variables. The resulting data sets enabled us to identify and characterize health assets with the potential to enhance social connections among older adults registered within health jurisdictional areas from 2015 to 2022. In addition, this approach allowed us to examine area-specific registration trends for health assets, showing the use of the Assets and Health platform developed as part of a public health strategy in Catalonia. We provided detailed explanations of concepts, steps, and the code used, and included supplementary information to facilitate the replication of the steps, attempting to familiarize novice readers with these techniques.

### Applications of the WeTMS Method

Our method provides a tool for researchers interested in developing new contextual variables when data are scarce or difficult to obtain using traditional means. Researchers in fields such as public health, nursing, and social epidemiology who study the impact of emerging health and social phenomena on health outcomes and determinants of health can benefit from this method. A practical example of an emerging social determinant of health, such as precarious employment [63], can consist of applying the web-scraping steps to obtain website data from employment portals, text mining to analyze posts, identifying precarious job offers, and spatial overlay analysis to locate them into geographic areas and study the effect on population outcomes using multilevel modeling. Researchers and program evaluators in health services research and implementation science can use this method to obtain data to conduct descriptive analyses explaining policy adoption within jurisdictional or geographic areas, following the research case outlined in this study.

A key feature of this method is that its steps can be implemented in sequence or independently, depending on the research goals. For example, researchers interested in generating new variables from text data without locating them in specific geographic areas can follow steps 1 to 6, which involve web scraping, text processing, and mining. If a data set is already available and researchers want to group the data by geographic settings, they can follow steps 7 and 8, which involve overlay spatial analysis.

### Challenges and Limitations

There are challenges with this method that can limit its feasibility and application. In our example, extracting comparable data from multiple URLs was feasible because the websites associated with the Assets and Health platforms had similar HTML structures. The consistent placement of targeted information across websites simplifies the complexity of the web-scraping program. Thus, the attached web scraper code is only suitable for single or multiple websites with a limited number of distinct HTML structures (eg, forums, social media, and employment portals). Studies in which the target data are spread over different websites with varied designs require more advanced programming [11].

A key step in the process—the creation of topic-specific dictionaries for the classification of observations—necessitates a deep understanding of the field and the terminology used in the data. Overall, the rule-based classifier demonstrated high accuracy. However, some variables, such as *activity type*, showed a higher rate of errors, in part because the dictionaries used to classify them contained only high-frequency words found in the titles and descriptions. Thus, our experience suggests that manual verification of new variables and categories by researchers with a comprehensive understanding of the data and subject matter is essential to ensure data validity before statistical analysis. However, this can be unfeasible for large data sets. In such scenarios, the impracticality of manual verification may necessitate the use of complex machine learning classifiers, presenting a trade-off in the confidence of the data that potentially compromises the robustness of the resulting variables [52].

Spatial overlay analysis effectively localizes health assets to their respective health jurisdictions, facilitated by the acquisition of complete addresses during the web scraping phase and the availability of a high-quality polygon map for analysis. Geospatial maps can be obtained from government agencies, nonprofit organizations, and commercial providers. If maps are unavailable, they can be created using accessible satellite imagery [64]. However, the necessity for location-specific data (eg, addresses, postal codes, and cities) for each observation to generate contextual-level variables limits the range of suitable data sources available to researchers.

Ethical and data protection considerations are important. Web scraping is typically permitted when data are publicly accessible and not subject to international legislation concerning personal data, trademarks, copyrights, or private information [30]. Automatic extraction of internet data might be unfeasible if the data are not publicly available or if a website’s terms of service restrict automated collection and analysis [65]. Researchers may consult ethics bodies to ensure that the methodology adheres to ethical standards when dealing with sensitive topics and personal information, even when relying on publicly available sources.

In addition to these challenges, the method and data sets that it produces have limitations. The complexity of these steps requires introductory technical knowledge. Thus, we have provided detailed explanations and supplementary information that can support researchers, as they familiarize themselves with the steps. We anticipate that the compendium of concepts, code, software packages, and references gathered from trustworthy sources will serve as a resource for those interested in these techniques.

Another limitation is the bias associated with the classifier system. The development of classification systems inherently relies on the subjective judgment of researchers. This can result in misclassifications, particularly those related to assumptions about race, gender, or social exclusion factors, especially within machine learning classifiers [66]. It is advisable for researchers to engage in a reflexive process, carefully considering their assumptions in the definition and selection of dictionary words and to critically evaluate how these decisions may influence the investigation [67].

Finally, although the data sets generated are robust for descriptive analysis, researchers should proceed with clearly defined assumptions when using new context-level variables in statistical analyses, particularly in multilevel modeling or ecologic studies that aim to draw inferences. Although we successfully compiled 2 data sets of health assets from targeted websites, their comprehensiveness and accuracy in reflecting all identified assets across BHAs remain unknown. It is possible that some local organizations in BHAs were more or less likely to register assets on Assets and Health websites, influenced by context-specific factors such as management support and training on the platform [68]. If feasible and ethical, it would be advisable for researchers to triangulate data to validate the data sets, thus verifying web-scraped data with secondary sources or direct inputs from stakeholders [69].

### Conclusions

The sequential use of WeTMS enabled the efficient creation of data sets of health assets registered with the Assets and Health websites in Catalonia, Spain, which aimed to enhance the social connections of older adults in local health jurisdictions. Our descriptive analysis demonstrated the usefulness of the data sets in exploring the characteristics of contextual variables, as well as in understanding temporal patterns and spatial distributions.

Contextual-level variables generated via WeTMS may also be used in hierarchical analyses to evaluate the impact of contextual factors on health outcomes when more robust sources, such as census data, are not available. Adherence to data protection standards and ethical considerations should also guide this process. Although WeTMS has potential value for multiple research disciplines, it presents challenges and limitations, including the need for internet data sources to have comparable structures, a dependence on location data, the potential lack of representativeness in website content, the requirement for technical expertise, and a significant time investment for manual verification.

## Appendix

Multimedia Appendix 1 Additional details in step 2: identifying hidden URLs, finding HTML elements, and further references.

Multimedia Appendix 2 Detailed explanation of Python libraries and web scraper code.

Multimedia Appendix 3 Definitions of new variables and categories for text classification.

Multimedia Appendix 4 Extended descriptive analysis of the type, target population, focus, cost, format, and duration of health assets.

## Abbreviations

BHA: basic health area
CRS: coordinate reference system
SDOH: social determinants of health
WeTMS: web scraping, text mining, and spatial overlay analysis

## Data Availability

The data sets generated and analyzed during this study are available from the corresponding author upon reasonable request.
