# Supplementary material for: Generating Contextual Variables From Web-Based Data for Health Research: Tutorial on Web Scraping, Text Mining, and Spatial Overlay Analysis
Source: JMIR Public Health Surveill. 2024 Jan 8;10:e50379. doi: 10.2196/50379 (PMC10804251; doi:10.2196/50379)
Supplement: Multimedia Appendix 1 [file publichealth_v10i1e50379_app1.docx]

**Additional details on Step 2: Identifying hidden URLs, finding HTML elements and further references.**

***Identifying hidden URLS: Network inspector***

The network inspector is located on a browser’s DevTools (F12 key, network tab). With the network inspector window open, click on the object of interest (eg, hyperlink opening a new website containing the information of interest). In our study, the objects of interest were the hyperlinks opening the websites containing full descriptions of each health asset located in the search engine of the Asset and Health platform (Image S1, letter a). Detailed information about the request will appear, starting with the hidden URL of the website in which the health asset description was stored (Image S1, letter b).

Image S1. Using the network inspector DevTool to identify hidden URLS.


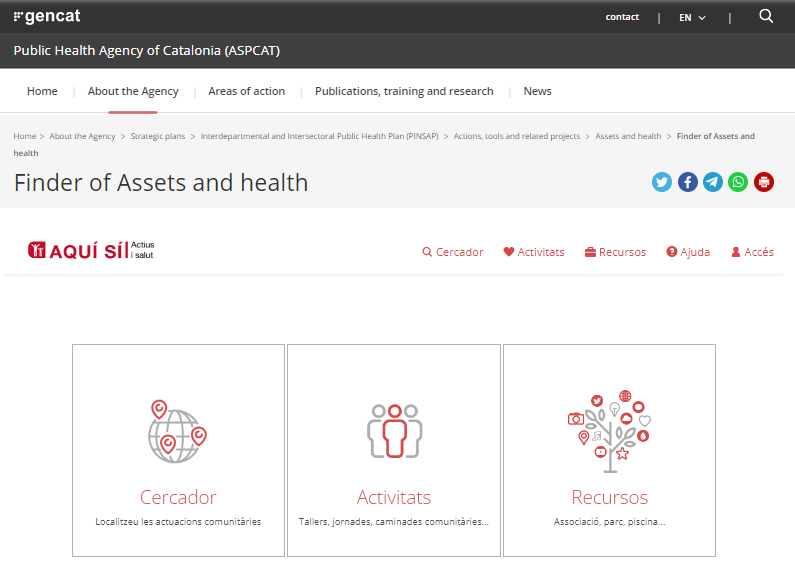

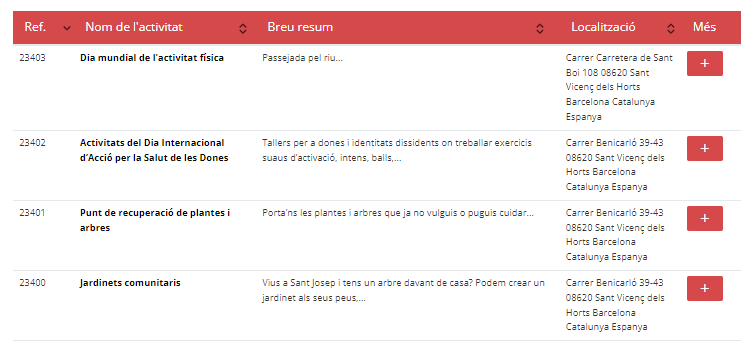


a

**Image 1.** Network inspector on Google Chrome.


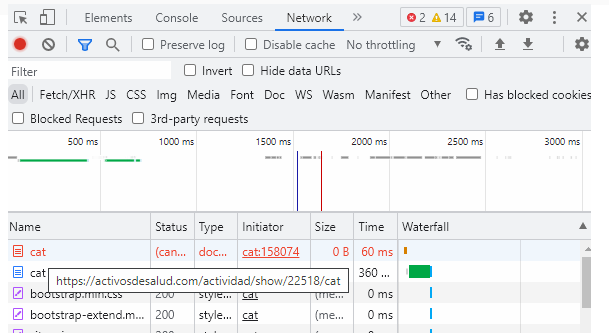


b

***Navigating the HTML code to identify relevant HTML elements***

The HTML code is organized in lines named HTML elements. Each element is a building block of a web page which contains a piece of information., listed as a line in the code tree. HTML elements are opened and closed by a *“tag”* denoting the type of content, and have specific attributes. The *“id”* attribute specifies a unique id for an HTML element which cannot be repeated. The *“class”* attribute is present in some elements which might not have an “id”, giving style to the element. In conclusion, the researcher needs to identify what HTML elements contain the information of interest, their “tags”, “id” and “class” to develop a web scraping program targeting such elements.

Image S2 shows an example of a website accessed through a hyperlink in the main Asset and Health platform containing a full description for a health asset activity. As in the network inspector, the HTML code is accessible through the bowser developer tools (F12 key, elements tab). To identify the specific HTML elements containing the target information, we used the elements inspector, accessed through the shortcut Ctrl+Shift+C (Image S2, a). Once the element inspector is activated, clicking on any element on a web page will highlight the corresponding HTML element code in the developer tools (Image S2, b). As appears in Image S2, for each website containing full descriptions of health assets type activity, we scraped the activity title (1), description (2), target population (3), location (8), date of registration (4), activity status (5), cost (6), duration (9), and activity topics (7), which are general themes associated to each activity.

Image S2. Finding the HTLM elements in each website containing target information.

a


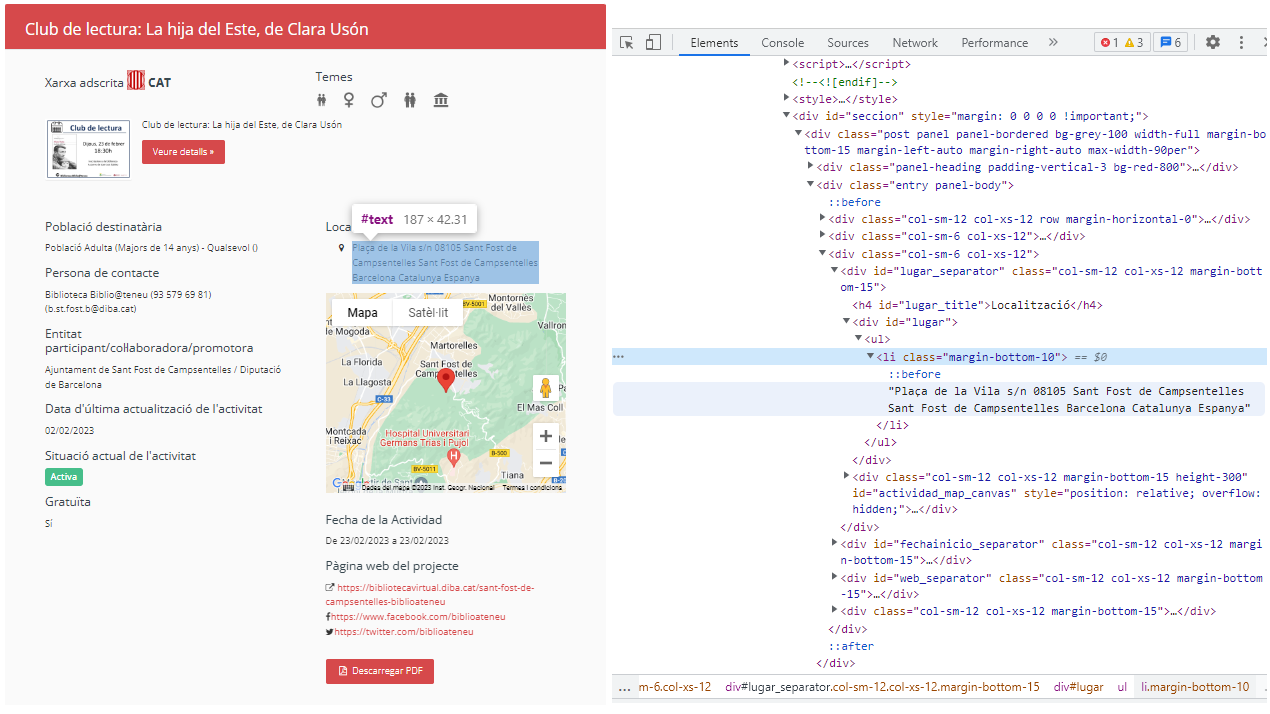


1

2

3

4

5

6

7

8

9

b

**Image 2.** HTML tree of a targeted health asset URL.

***Relevant resources to understand the HTML tree and the attributes:***

- vanden Broucke S, Baesens B. Practical Web Scraping for Data Science: Best Practices and Examples with Python. 1st edition. New York, NY: Springer Science Business Media; 2018. ISBN-13 (pbk): 978-1-4842-3581-2. Chapter 2 & 3. <https://link.springer.com/book/10.1007/978-1-4842-3582-9>
- Munzert S, Rubba C, Meiner P, et al. Automated Data Collection with R: A Practical Guide to Web Scraping and Text Mining. 1st edition. West Sussex, UK: John Wiley & Sons, 2015. ISBN: 9781118834817 (pp. 15-40).
- W3 Schools. HTML Elements. 2023. URL: <https://www.w3schools.com/html/html_elements.asp>
- W3 Schools. HTML Tags. 2023. URL: <https://www.w3schools.com/tags/>

***Relevant resources on extracting and parsing URL source data***

Installing Python and PyCharm:

- Max Teaches Tech. Install PyCharm & Python on Windows 10. [video]. Youtube. Published January 23, 2021. URL: https://www.youtube.com/watch?v=XsL8JDkH-ec
- vanden Broucke S, Baesens B. Practical Web Scraping for Data Science: Best Practices and Examples with Python. 1st edition. New York, NY: Springer Science Business Media; 2018. ISBN-13 (pbk): 978-1-4842-3581-2. Chapter 1.

Introductory resources to web scraping:

- Wu S. Web Scraping Basics. 2020 [website]. URL: <https://towardsdatascience.com/web-scraping-basics-82f8b5acd45c>
- Munzert S, Rubba C, Meiner P, et al. Automated Data Collection with R: A Practical Guide to Web Scraping and Text Mining. 1st edition. West Sussex, UK: John Wiley & Sons, 2015. ISBN: 9781118834817 pp. 101-148 & pp. 219-294

Introduction to the libraries requests, beautifulsoup, pandas

- Shafer C. Python Tutorial: Web Scraping with BeautifulSoup and Requests. [video]. Youtube. Published November 8, 2017. URL: https://www.youtube.com/watch?v=ng2o98k983k
