# Supplementary material for: Generating Contextual Variables From Web-Based Data for Health Research: Tutorial on Web Scraping, Text Mining, and Spatial Overlay Analysis
Source: JMIR Public Health Surveill. 2024 Jan 8;10:e50379. doi: 10.2196/50379 (PMC10804251; doi:10.2196/50379)
Supplement: Multimedia Appendix 2 [file publichealth_v10i1e50379_app2.docx]

**Detailed explanation of the Python libraries and the web scraper code.**

Python is a widely used object-oriented programming language suitable for web scraping, with an extensive user community and support resources. Although multiple libraries allow web scraping, we propose the “requests”, “BeautifulSoup4”, and “pandas” libraries, which are suitable for most scenarios. The “requests” library sends HTTP (Hypertext Transfer Protocol) requests from Python to access a resource on a server through an URL. Once the webpage is accessed, the “Beautiful Soup 4” library allows navigating, searching, and extracting the HTML elements. Finally, the “pandas” library enables automatic organization of the scraped HTML content into tabular data composed of columns and rows and creating a .csv file.

Below is the Python code developed for the study, which was used to automatically scrape health assets data from activity and resource web pages. The code for both types of assets (activities and resources) was similar, except for the number and type of information scraped, the variables returned, and the URLs. Below is a brief explanation of each section of the code.

| **import** requests  **from** bs4 **import** BeautifulSoup **as** bs **import** pandas **as** pd  *# This section of the code imports the required libraries* |
| --- |
| **def** getIfExists(elem):  **return** elem.text.strip() **if** elem **else None**  *# This function takes an HTML element as an argument and returns the text content. If the element does not exist, the function returns None, avoiding errors when trying to access non-existent elements. This function is applied later when parsing HTML elements.* |
| **def** getDataFromUrl(url):  r = requests.get(url)  **if** isinstance(r.text, str) **and "An Error Occurred" in** r.text:  **return None**  *# This function takes a URL as an argument and uses the requests library to send a request to that URL. The function then checks if the response from the server is a string and whether the string contains the text 'An Error Occurred'. This handles potential errors that may occur when accessing a webpage that does not exist. The function is applied later in the web crawler portion of the code.* |
| title = getIfExists(soup.find(**"h2"**)) description = getIfExists(soup.find(**'div'**, {**'id'**: **'descResumen'**})) populationTarget = getIfExists(soup.find(**'div'**, {**'id'**: **'diana'**})) location = getIfExists(soup.find(**'div'**, {**'id'**: **'lugar'**})) organizations = getIfExists(soup.find(**'div'**, {**'id'**: **'centro'**})) updatedOn = getIfExists(soup.find(**'div'**, {**'id'**: **'fechaact'**})) typeAsset = getIfExists(soup.find(**'div'**, {**'id'**: **'sitatual'**})) isFree = getIfExists(soup.find(**'div'**, {**'id'**: **'gratuita'**})) **try**:  duration = getIfExists(soup.find(**'div'**, {**'id'**: **'fechainicio'**})) **except** Exception **as** e:  *# print(e)* duration = **None**  categories_asset = soup.find_all(**'img'**, class_=**'float-left cateimages'**) categories = [] **for** i **in** categories_asset:  temp = i[**'title'**]  categories.append(temp)  *#This block of code utilizes the Beautiful Soup library to extract data from specific HTML elements on a webpage. The “getIfExists” function is invoked with various arguments to locate elements with specific HTML tags and attribute values. The text content of each element is then extracted and assigned to the corresponding variable.* |
| **return** title, description, populationTarget, location, organizations, updatedOn, typeAsset, isFree, **","**.join(categories) **if** categories **and** len(categories) **else None**, duration  *# This line of code returns a tuple, an object that can store multiple items, containing extracted data from the webpages. It also defines how to store variables with multiple values, such as categories, in a comma-separated list if those values exist.* |
| df = pd.DataFrame(columns=[  **"title"**,  **"description"**,  **"populationTarget"**,  **"location"**,  **"organizations"**,  **"updatedOn"**,  **"typeAsset"**,  **"is_free"**,  **"categories"**,  **"time_activity"**, ])  *#This part of the code creates a new empty Pandas data frame object “df” with column names specified in the list. This data frame will store the extracted information from multiple webpages, with each row representing a different health asset scraped and the columns representing the variables extracted.* |
| **for** index **in** range(0, 25000):  currentActivity = getDataFromUrl(**f"https://activosdesalud.com/actividad/show/{**index**}/cat"**)  **if** currentActivity **is None**:  **continue**  df.loc[len(df.index)] = currentActivity  df.to_csv(**"FinalAct231222.csv"**, index=**False**)  *#The final segment of the code encompasses the web crawler, which is a loop iterating over a range of numbers from 0 to 25,000. These numbers correspond to the reference numbers of the health assets. In each iteration, the “getDataFromUrl()” function, defined earlier, is invoked, constructing a request to a URL that includes a fixed segment and the current index value. Subsequently, this function parses the previously described HTML elements in the HTML tree and saves the data into a CSV file at the conclusion of each loop iteration.* |
