# Supplementary material for: Generating Contextual Variables From Web-Based Data for Health Research: Tutorial on Web Scraping, Text Mining, and Spatial Overlay Analysis
Source: JMIR Public Health Surveill. 2024 Jan 8;10:e50379. doi: 10.2196/50379 (PMC10804251; doi:10.2196/50379)
Supplement: Multimedia Appendix 3 [file publichealth_v10i1e50379_app3.docx]

**Definitions of new variables and categories for text classification.**

Supplementary Table S1 and Table S2 include definitions for the variables and categories that guided the creation of topic-specific dictionary words and text classification during step 5: Defining dictionaries and categorizing text.

Table S1***.*** Definitions for new variables and categories to classify health assets of type activity.

| **New variables/***Categories* | **Definitions and examples** |
| --- | --- |
| **Activity type** | This variable categorizes health assets related to activity type based on their constituent components and mechanisms of action, which may enhance social connections among older adults. This variable contains categories proposed in previous studies [46,48,49] to classify interventions to reduce social isolation and loneliness among older people. |
| *Leisure and skill development* | Leisure activities are recreational activities or hobbies, such as gardening, travelling, playing, or dancing. Skill development activities, on the other hand, are activities focused on acquiring new skills or improving existing ones, such as computer programming course, cooking classes, or learning a new language [46]. |
| *Physical activity* | Group activities involving exercise or exercise plus leisure, such as group walking, adapted physical exercise for older adults [48]. |
| *Social facilitation* | Social facilitation activities are designed to promote social interaction among individuals. Often involving group-based activities such as charity-funded friendship clubs, reading groups, shared interest group, day care centers, and friendship enrichment programs [46]. |
| *Psychological therapies* | These types of activities employ therapeutic techniques administered by trained health professionals or therapists. A key aspect is the use of facilitated group activities. Examples include humor therapy, mindfulness and stress reduction, reminiscence group therapy, and cognitive enhancement and social support interventions [46]. |
| *Awareness campaigns* | Activities designed to enhance knowledge and understanding of health and social issues concerning older adults, including social connection problems such as social isolation and loneliness, both among healthcare professionals and the general public [49]. |
| *Health and social care* | Group activities where healthcare and social professionals provide support to older population either through direct intervention or education on health or social topics [46]. Includes health education (e.g., group seminars for older adults with diabetes), health promotion and prevention (e.g., group intervention to prevent falls), social care (e.g., literacy for older migrants). |
| *Befriending* | Befriending activities refer to a type of social facilitation typically involving one-to-one interactions with volunteers sustained over time (e.g., companion programs) [46]. |
| *Animal Based* | Activities involving animals which focused mainly on animal-assisted therapy [46]. |
| **Format** | This variable categorized health assets of type activity depending on the number of individuals targeted [49]. |
| *Group* | Group activities are directed to two or more individuals. Group activities may be led by a health professional or instructor, such as a fitness class or group therapy session, or be more informal, such as social gatherings, a walking club, or a reading club. |
| *Individual* | These activities are directed to one individual. Examples include individual therapies, and one-to-one companionship activities such as befriending. |
| **Focus** | This variable categorizes activities based on whether their primary objective is to address social connection issues, such as loneliness or social isolation, or whether social connection is a potential outcome that may arise from the social nature of the activity but is not the primary goal [49]. |
| *Direct* | Health assets activities with “direct” focus are interventions that specifically aim to address social connection issues such as social isolation, loneliness, or related constructs [49]. |
| *Indirect* | Activities with an “indirect” focus do not explicitly aim to address social isolation or loneliness but can still have a significant impact on an individual's perceived or actual social connections. Examples include participating in a group health-focused physical activity program, which can lead to a reduction in social isolation or loneliness due to the program's social nature [49]. |
| **Age** | This variable categorizes de health assets in age groups based on the target population. |
| *Children, Youth, Adults, Older adults* | These categories were used to classify activities based on standard age groups proposed by WHO-UN [50]: children (0-14 years), youth (15-24 years), adults (25-59 years), and older adults (over 60 years). |
| *General Population*  *Minors unspecified*  *Adults unspecified* | The activity is designed for the overall community or region without a specific focus on any age subpopulation.  The categories of minors and adults unspecified categorize activities based on whether they target individuals under or above 18 years old, without specifying a particular age group. |
| **Gender** | This variable categorizes health assets based on the gender of the target population, based on common gender classification [51]. |
| *Women*  *Men*  *Non-Binary*  *Any* | Individuals whose reported gender is female.  Individuals whose reported gender is male.  Individuals whose reported gender is not exclusively male or female.  Activity designed without a focus on any specific gender. |
| **Vulnerable population** | Categorizes health assets based on whether a specific vulnerable population is being targeted. |
| *Migrants* | First generation migrants who moved from another country, including refugees. |
| *Caregivers* | Informal caregivers, often family members that provide unpaid care to older and dependent persons. |
| *Addictions* | Individuals experiencing substance abuse, including alcohol, illegal drugs and tobacco. |
| *Physical diseases* | Conditions that affect the organs, tissues, or systems of the body, leading to impaired normal function. Examples include cancer, cardiovascular diseases, diabetes, arthritis, and musculoskeletal conditions. |
| *Risk social exclusion* | Individuals with higher likelihood of being excluded from participating in social, economic, and cultural aspects of society. |
| *Mental diseases* | The category mental diseases encompass mental disorders (e.g., depression, anxiety) and neurodegenerative diseases (e.g., dementia), affecting an individual's thinking, mood, and behavior, impairing daily functioning. |
| *All* | Activity targeting any individual regardless of their vulnerable status. |

Table S2. Definitions new variables and categories to classify health assets of type resource.

| **New variables/***Categories* | **Definitions and examples** |
| --- | --- |
| **Resource type** | This variable categorizes health assets of type resources based on their physical and organizational characteristics, as well as their intended purpose or type of use. The definitions are created by the research team to encompass the different types of resources found during the text analysis. |
| *Municipal natural and green space* | Publicly owned natural or green areas, such as parks or gardens, that are managed by local government. |
| *Health institution* | Organizations that provide healthcare services, such as primary care centers, public health services, or hospitals. |
| *Social welfare institution* | Organizations that provide social services and promote social integration to individuals and families in need. |
| *Education institution* | Institutions that provide educational services, such as schools, including continuing education centers for the older population. |
| *Patient advocacy group* | Non-profit organizations that advocate for the rights and interests of patients with specific medical conditions. |
| *Charitable & voluntary organization* | Non-profit organizations that provide charitable services or volunteer opportunities to support social causes or community needs. |
| *Faith-based organization* | Organizations that are based on religious or spiritual beliefs. |
| *Parents/family schooling associations* | Organizations formed by parents and family members to support and improve educational programs and services for their children. |
| *Public Library* | A publicly funded institution that provides free access to books, media, and other educational resources. |
| *Civic center* | A publicly owned facility that provides community services and programs, such as recreational activities, cultural events, or social services. |
| *Sports institution* | Organizations that provide sports and physical fitness programs and services, such as gyms or sports clubs. |
| *Leisure & cultural association* | Non-governmental groups formed by civil society members that promote cultural or leisure activities. |
| *Neighborhood association* | Organizations formed by residents of a neighborhood to address community issues and promote community well-being. |
| *Cultural institution* | Organizations that promote cultural activities and preserve cultural heritage, such as such as museums or theaters. |
| **Focus, Age, Gender, Deprived population** | The same categories and definitions described above are shared between activities and resources. |
