# Supplementary material for: Generating Contextual Variables From Web-Based Data for Health Research: Tutorial on Web Scraping, Text Mining, and Spatial Overlay Analysis
Source: JMIR Public Health Surveill. 2024 Jan 8;10:e50379. doi: 10.2196/50379 (PMC10804251; doi:10.2196/50379)
Supplement: Multimedia Appendix 4 [file publichealth_v10i1e50379_app4.docx]

**Extended descriptive analysis of type, target population, focus, cost, format, and duration for health assets.**

Table S3. Descriptive analysis of health assets based on target population.

| **New categories for**  **target population** | **Activities (N=5022**) | **Resources (N=4524**) |
| --- | --- | --- |
|  |  |  |
| **Age** | **n(%)** | **n(%)** |
| Older adults | 1235 (24.6) | 207 (4.6) |
| Adults unspecified | 2648 (52.7) | 100 (2.2) |
| General Population (all Ages) | 1139 (22.7) | 4217 (93.2) |
| **Gender** |  |  |
| Only Women | 143 (2.8) | 69 (1.5) |
| Only Men | 11 (0.3) | 5 (0.1) |
| No binary | 1 (0.0) | 9 (0.2) |
| Any gender | 4867 (96.9) | 4441 (98.2) |
| **Vulnerable population** |  |  |
| Substance abuse | 15 (0.3) | 19 (0.4) |
| Migrants | 18 (0.3) | 19 (0.4) |
| Risk social exclusion | 33 (0.6) | 80 (1.8) |
| Caregivers | 80 (1.6) | 14 (0.3) |
| Mental diseases | 252 (5.1) | 97 (2.1) |
| Physical diseases | 280 (5.6) | 132 (2.9) |
| All population | 4344 (86.5) | 4163 (92.1) |

Table S4. Descriptive analysis of type and characteristics of activities targeted at older adults with potential to enhance social connections. N=5022.

| **New categories for activities characteristics** | **n (%)** |
| --- | --- |
|  |  |
| **Type of activity** |  |
| Leisure and skill development | 1,844 (36.7) |
| Physical activity | 1,561 (31) |
| Psychological therapies | 655 (13.0) |
| Health and social care | 448 (9.2) |
| Social facilitation | 425 (8.4) |
| Awareness campaigns | 62 (1.2) |
| Befriending | 20 (0.4) |
| Animal based | 7 (0.1) |
| **Focus** |  |
| Direct | 219 (4.4) |
| Indirect | 4803 (95.6) |
| **Format** |  |
| Group | 5000 (99.6) |
| Individual | 22 (0.4) |
| **Free** |  |
| Yes | 2862 (56.9) |
| No | 1680 (33.5) |
| Unregistered | 480 (9.6) |
| **Duration** |  |
| < 1 week | 852 (16.9) |
| 1 week- 1 month | 236 (4.7) |
| 1-3 months | 975 (19.5) |
| 3-12 months | 479 (9.5) |
| >12 months | 17 (0.3) |
| Not registered | 2463 (49.1) |

Table S5. Descriptive analysis of type and characteristics of resources targeted at older adults with potential to enhance social connections. N=4524.^a^

| **New categories for resources characteristics** | **n (%)** |
| --- | --- |
|  |  |
| **Type of resource** |  |
| Charitable & voluntary organization | 52 (1.1) |
| Cultural institution | 265 (5.9) |
| Faith-based organisation | 29 (0.6) |
| Leisure & cultural association | 878 (19.4) |
| Municipal natural and green space | 782 (17.3) |
| Parents schooling associations | 31 (0.7) |
| Patient advocacy group | 146 (3.2) |
| Social welfare institution | 140 (3.1) |
| Sports institution | 226 (5.0) |
| Public library | 836 (18.5) |
| Neighbourhood association | 24 (0.5) |
| Health institution | 595 (13.1) |
| Education institution | 42 (0.9) |
| Civic center | 479 (10.6) |
| **Type of resource by area of action** |  |
| Cultural | 2782 (61.5) |
| Economic | 45 (1.0) |
| Environment | 786 (17.4) |
| Health | 749 (16.6) |
| Multiple social activity | 142 (3.1) |
| Politic | 21 (0.5) |
| **Focus** |  |
| Direct | 25 (0.6) |
| Indirect | 4500 (99.4) |

^a^ Data on the format and duration of resources was not available due to the resources permanent and open format Additionally, cost data for resources was not available.
